# Supplementary material for: Bacterial vitamin B6 is required for post-embryonic development in C. elegans
Source: Commun Biol. 2024 Mar 26;7:367. doi: 10.1038/s42003-024-05992-2 (PMC10966028; doi:10.1038/s42003-024-05992-2)
Supplement: Supplementary file 2 — Supplementary information [file 42003_2024_5992_MOESM2_ESM.pdf]

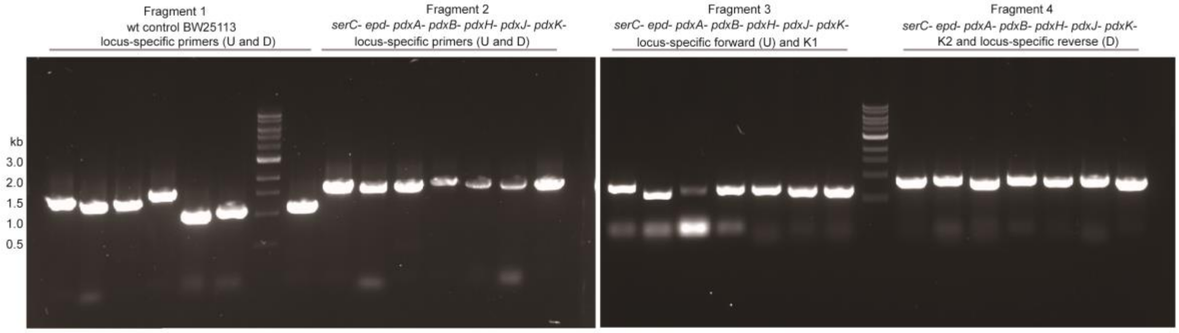
Fragment 1
wt control BW25113
locus-specific primers (U and D)
Fragment 2
serC- epd- pdxA- pdxB- pdxH- pdxJ- pdxK-
locus-specific primers (U and D)
Fragment 3
serC- epd- pdxA- pdxB- pdxH- pdxJ- pdxK-
locus-specific forward (U) and K1
Fragment 4
serC- epd- pdxA- pdxB- pdxH- pdxJ- pdxK-
K2 and locus-specific reverse (D)
kb
3.0
2.0
1.5
1.0
0.5

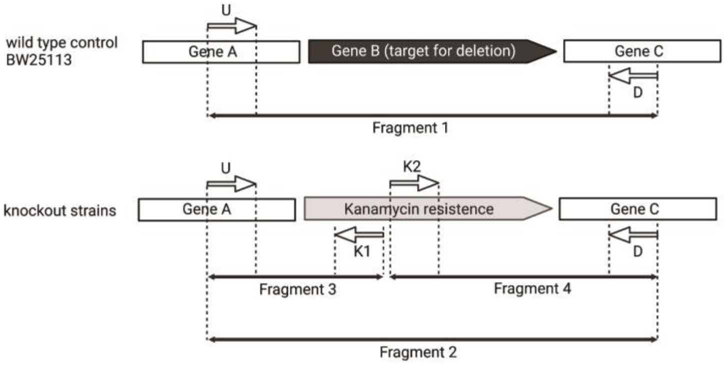
wild type control
BW25113
U
Gene A
Gene B (target for deletion)
Gene C
D
Fragment 1
knockout strains
U
K2
Gene A
Kanamycin resistence
Gene C
K1
D
Fragment 3
Fragment 4
Fragment 2

Supplementary Figure 1 PCR verification was performed to confirm the kanamycin cassette insertion and primer design. The wild type control strain BW25113 and 7 knockout strains were subjected to PCR amplification, and the resulting products were analyzed on a 1% agarose gel. The kan cassette insertions into the genome were validated by culturing in kan-LB media and by performing PCR with neighboring gene locus-specific (U and D) primers in combination with kanamycin (K1 and K2) primers. The NCBI Nucleotide database was used to confirm the K-12 wild-type gene size.

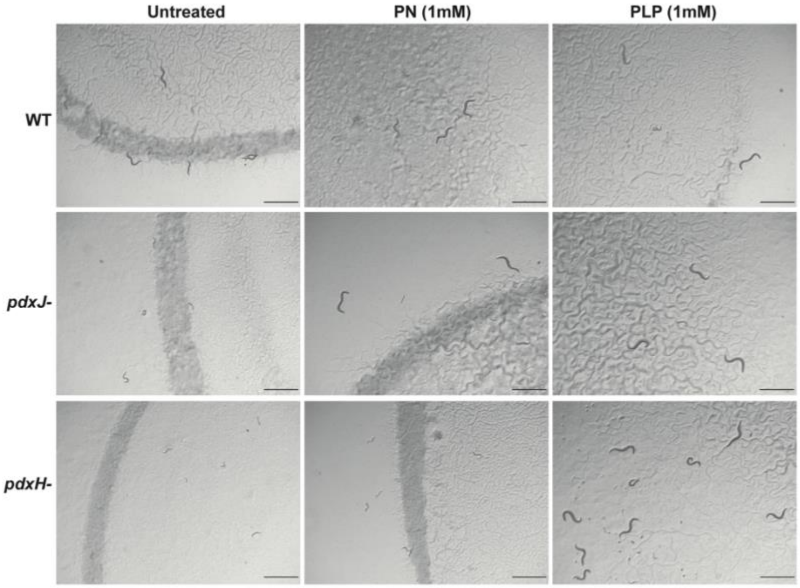
Untreated
PN (1mM)
PLP (1mM)
WT
pdxJ-
pdxH-

Supplementary Figure 2 Representative images of worms fed on 0.2µl *E. coli pdxJ* or *E. coli pdxH* mutants together with 150µl *L. plantarum* and supplemented with either the intermediate pyridoxine (PN) or the final metabolite pyridoxal 5′-phosphate (PLP). Images were taken 3 days after synchronized L1 worms were placed on the plates. Scale bar, 1000 µm.

Keio Gene Name	EcoCyc Gene Name	Forward (5' -> 3')	Reverse (5' -> 3')
epd	epd	agtcgcagtttgcgacaggtaagg	tttgcgccttgtttcagggcca
serC	serC	tcgggcaaaacgggtgacctga	gccagcaataaagcgcggttagaa
pdxA	pdxA	ccggttcactcttcattcggct	agaccggggccgatttcgaccat
pdxB	pdxB	gataaaggctgattgcccgtgc	cttaccaccaaagcgcagttgt
pdxJ	pdxJ	aacgcacgggaatttcctgacg	aacgccagaccattgcggatcc
pdxK	pdxK	gcagttaacttcgcgtaaacgggg	cctgactccggttgttatctccaa
pdxH	pdxH	aaacagggcatttcagcctctggc	gcccccgctcttgcaattgttt
K1	KanR		CAGTCATAGCCGAATAGCCT
K2	KanR	CGGTGCCCTGAATGAACTGC	

Supplementary Table 1 Primers used in this study.
